# The Measurement Problem in the Thermodynamics of Black Holes

**DOI:** 10.3390/e28070808

**Published:** 2026-07-15

**Authors:** Jeroen Schoenmaker

**Affiliations:** Center for Engineering, Modelling and Applied Social Sciences, Federal University of ABC (UFABC), Santo André 09280-560, SP, Brazil; jschoenmaker@gmail.com or jeroen.schoenmaker@ufabc.edu.br

**Keywords:** thermodynamic entropy, information entropy, hysteresis, noise, measurement, signal-to-noise ratio, communication, black hole information paradox

## Abstract

This manuscript gives a solution to the black hole information paradox by bringing to the debate a fundamental aspect of information science: the process of measurement by a receiver. Bekenstein and Hawking established the foundations of black hole thermodynamics based on previous works of Brillouin and Szilard on information physics. In this work, we demonstrate that the relation between energy and information established in communication technology by Shannon and Landauer has not been adequately applied to black hole physics. As Landauer states, a computation process is closely akin to a measurement. Our argument is grounded on the physical concepts of measurement, signal-to-noise ratio, energy dissipation during the switching process in computation, and hysteresis loops. We give special attention to the role of noise and energy dissipation in the process of information transmission. We demonstrate that Szilard’s work fails to establish a connection between information and entropy in agreement with the works of Landauer and Shannon. We also demonstrate that a quantum state cannot be directly equivalent to a unit of information. The entropy and temperature attributed to black holes are questioned, and a solution to the black hole information paradox is provided. Similarly to what happens with Maxwell’s demon, the black hole information paradox is “exorcised” once we account for the process of measurement and information processing.

## 1. Introduction

Consider the situation: Alice sends a message to Bob. The message package consists of a square piece of paper with an “8” written on it, an alpha particle, an envelope with a penny inside in which the head is directed to the face of the envelope, an apple, a photon of wavelength λ, and a dice. According to the current paradigm, if Bob is a black hole, the package corresponds to information falling into it, and physicists could discuss if the information is lost or not. On the other hand, if Bob is a person, from the point of view of Shannon’s theory of information, there is no communication established between Alice and Bob. How could he detect the photon and the alpha particle? Bob rotates the piece of paper and is not sure if it is an “8” or an “∞”, or even another symbol from an alphabet unknown to him. Is the apple part of the message? Is the envelope a payment or part of the message?

The situation just described illustrates how Shannon’s mathematical theory of communication is distinct from black hole thermodynamics. Many physicists may argue that they are essentially the same. In this study, we emphasize that it is important to analyze how information entropy went from communication technology to black holes. We argue that information entropy is mishandled in the black hole realm. In order to sort this issue, we have to clarify and adequately place many fundamental concepts. Instead of analyzing whether the information is preserved or not in the black hole, we take a step back and make an in-depth analysis on the foundations of black hole thermodynamics in order to verify whether the information paradox has a sound physical justification in the first place.

Some physicists may reason that the message Alice sent to Bob could result in a communication. We sustain that, in the framework of the theory of information established by Shannon and still applied to current data processing and communication technology, it could not, indicating that the entropy of the black hole could not be calculated the way it was originally proposed by S. Hawking and J. Bekenstein. In this context, the transition of information entropy from communication technology to physics was based on the works of L. Szilard and L. Brillouin that we also analyze here.

The concept of entropy has been introduced in thermodynamics by works of S. Carnot and R. Clausius. Afterwards it was established statistically by L. Boltzmann and J. W. Gibbs, relying on the evaluation of accessible states of the physical system under consideration, that many physicists refer to as “the internal configurations of the system”. This principle works very well within a quantum–mechanical approach once is rather straightforward to account for the discrete states of a quantum system. Note however, that the quantum states of a system are not necessarily connected to the notion of information and this connection was not put forward by Boltzmann and Gibbs. Furthermore, in the case of black holes, we have to consider that there is no suitable quantum theory for a system of particles coupled by gravity. In this context, Bekenstein and Hawking used information entropy based on the works of Shannon and Brillouin. In this work, we discuss the applicability of information entropy to black hole physics.

The black hole information paradox has remained unsolved for more than 50 years. We may concede that paradoxes often arise when inconsistent assumptions are made. Moreover, very often fundamental scientific concepts are hard to define. Biologists struggle to have a good definition of life. Physicists struggle to have a good definition of energy. R. Feynman famously said “*It is important to realize that in physics today, we have no knowledge of what energy is*” [1] (Chapter 4, Page 2). As we demonstrate here, something akin happens with information. Perhaps the situation with information is somewhat more complicated than with energy.

Information is a complex concept to define, and is related to the notions of knowledge, understanding, sensing, recognizing, detecting, measuring, etc. Thus, in this manuscript we discuss how a physical system can be related to information on many different levels. Information science started as a theory of communication. How does a person understand, recognize, measure, and sense a word written on a page? How does a text extraction software understand, recognize, measure, and sense a word contained on an image that contains text? How does a particle of a gas understand, recognize, measure, and sense the presence of the walls of the container? How does a black hole understand, recognize, measure, and sense the fall of a particle through the event horizon? This is the kind of question that drives the path of this manuscript, from the mathematical theory of communication to the black hole thermodynamics. As we demonstrate, this path is based on sound physical concepts and important conclusions regarding the black hole information paradox is given.

At last, before we start our argumentation, it is worth preparing the reader for the following issue: there might be an objection regarding the abuse of language, when a phrasing such as “a particle recognizes the presence of a wall” appear in this manuscript, with the argument that a particle is not able to “know” anything. We argue that, in fact, this issue also belongs to the core of the discussion of this manuscript, for the founders of information physics rely on this kind of phrasing in order to establish their models likewise. And the discussion we put forward here is far from being subjective, but based on the solid physics of communication, measurement, data processing and statistical mechanics.

## 2. The Foundational Literature of Black Hole Thermodynamics

It is widely accepted that black hole thermodynamics was established with the publication of the manuscript entitled “*Black hole explosions?*” by Stephen Hawking in 1974, where he laid out the calculation for what became to be known as Hawking radiation [2]. This manuscript by Hawking departs from the premise that black holes have a temperature, based on two referenced manuscripts: one is a self-citation [3] and the other published by Bekenstein in 1973, entitled “*Black holes and Entropy*” [4]. This manuscript authored by Bekenstein is, in its turn, fairly self-referenced, among a few other citations to Hawking and close collaborators. However, when the manuscript attempts to base its thermodynamics claims, it does so strongly based on the following two works: “*A mathematical theory of Communication*” published by Shannon in 1948 [5] and “*Science and Information Theory*” published by Brillouin in 1956 [6]. It is worth noticing that Brillouin’s work is strongly based on the work of Szilard published in 1964 [7].

We argue that the above cited literature served as the backbone of the scientific knowledge in the foundation of the black hole thermodynamics. It was not our objective to exhaust the literature on information physics and on black hole thermodynamics. In this rather extensive manuscript, we focus our analysis on this selection of works in order to avoid unnecessary prolixity. We argue that our conclusions would not be altered in the case a wider literature would be considered.

## 3. Information, Data, Noise, Memory, Processing, Energy, and Entropy

As mentioned in the introduction, it is a commonplace that fundamental concepts are hard to define. This happens with the case of information, and very often the notions of understanding and meaning of information are included in the debate. Even in the technical realm, the issue is not completely settled. The communication technology community usually uses the term “information” whereas the data storage community prefers the term “data”. For most circumstances the two terms are interchangeable. However, some may argue that there is a difference related to the moment the message is understood. This is important because we have to be very clear in defining in which context and in what manner a message is understood. In this sense, the confusion on the notion of “understanding” can lead to several issues in a scientific debate. Consider the following example. A message in Dutch language is written and sent to the CEO of a company. The letter is first received by his secretary, who does not speak Dutch. He is able to recognize every letter written but is not able to understand the message. Afterwards, the secretary forwards the message to his boss, who speaks Dutch and reads it. Some may argue that, in the hands of the secretary, the message was data, but when the CEO reads it, it has become information. For most practical purposes, this distinction is irrelevant because the situation is consistent with the framework of the established information theory that accounts that the message was transmitted using a controlled set of variables under an established protocol, (the Dutch alphabet). As C. E. Shannon put it, “*These semantic aspects of communication are irrelevant to the engineering problem*” [5]. However, as we illustrated in the first paragraph of the introduction, the message needs to be somehow “understood”, to state it loosely, or “detected within a set of expected possible and recognizable values within an established protocol”, if we want to be a little more technical, in order to determine whether each of its components is a signal or is just noise. As we demonstrate in this manuscript, we have to resort to profound physical principles if we want to communicate in an effective and sophisticated communication technology.

### 3.1. Shannon and Landauer

The manuscript “*A Mathematical Theory of Communication*” is considered the birth of information theory [5]. In the context of Shannon’s work, information can be defined in terms of a quantity called entropy that is a measure of the number of possible choices of messages contained in a symbol, signal, transmitted message, or other information-bearing object. It is often expressed by the formula:

(1) $$H\;\left(X\right)=-\sum_{x\epsilon X}p\left(x\right)\;\log p(x),$$

where x is one possible outcome of the variable X and p(x) is the probability of the outcome x.

There is an anecdote saying that J. von Neumann suggested to Shannon that he should call the measure of information “entropy”, giving two justifications: first, the uncertainty function has been used in statistical mechanics under that name. Second, no one knows what entropy really is. The question whether the anecdote is true or not is not really relevant. What matters is that Shannon decided to name this quantity “entropy”, a term borrowed from thermodynamics that physicists agree is hard to grasp. More importantly, Shannon believed that correlation between information entropy and thermodynamic entropy was nothing more than an analogy.

**Figure 1:**
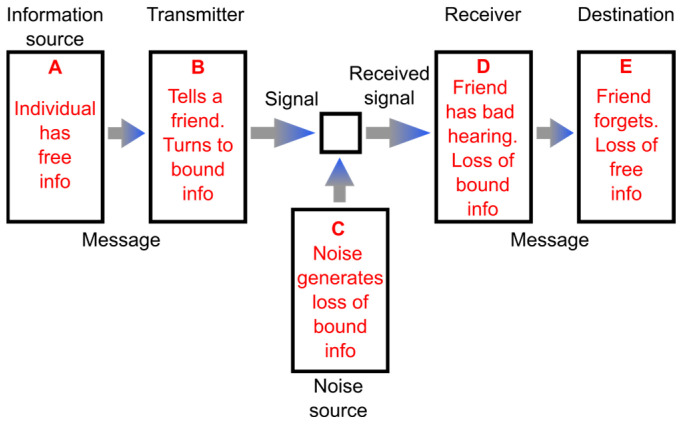
Merging of the communication process sketched by Shannon and Brillouin. In black, the well-known diagram of a general communication system proposed by Shannon. In red (inside the boxes), is a sketch of the Brillouin description of the process of communication between individuals in which he exemplifies the distinction of free and bound information, adapted to the Shannon diagram.

The diagram of a general communication system devised by Shannon can be seen in Figure 1, where the essential roles of noise and the receiver are evident.

In the first sentence of his treatise, Shannon states that the issue of signal-to-noise ratio was the main driver for the creation of his theory of communication. In fact, one of the main results of his work is the noisy-channel coding theorem. This matter is very important to settle and place in order to support the conclusions of this manuscript. The central question of the signal-to-noise issue is: how can we separate what is a signal from what is noise? This question is essentially related to another central question in theory of information: how can we know that the outcome of a variable is “this” and not “that”. If the variable is the alphabet, then we have to be able to measure (or detect) that the signal is an “a” and not any other letter with some degree of reliability. If it is a binary system, we have to measure that it is a “1” and not a “0”, and so forth.

In simple terms, ensuring that the message is transmitted according to a protocol and ensuring that the receiver detects mostly signals in a way that noise is suppressed to an acceptable standard, means that, at the engineering level, the receiver “understood” the message. This is how we bypass the semantics debate and are able to argue and formulate the issue of information technology solely in terms of the physical aspects of communication.

**Figure 2:**
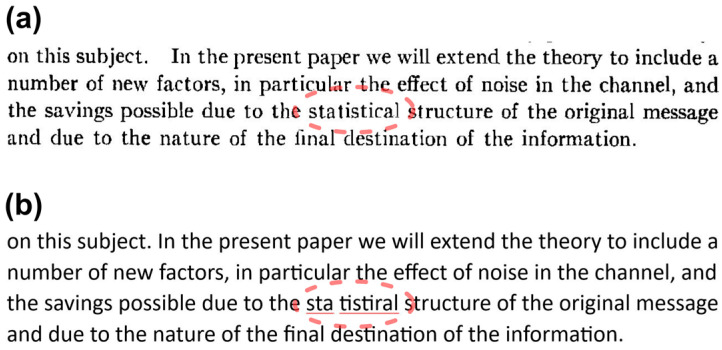
Snippet from the 1948 Shannon’s manuscript. (**a**) The image of the photographed document. (**b**) The wording obtained by a text extraction software. Note how the aging printed document resulted in a noisy photographed image. As a consequence, the word “statistical” was recognized by the software as two separated words and the letter “c” in was recognized as a letter “r”. This is a good example, within the material used for the preparation of this manuscript, on the relevance of each variable, in a communication system, to be recognizable and stable. Note that, by using the term “recognize” we are meaning that the software, to the engineering level, “understands” the message, avoiding the debate regarding semantics.

In order to avoid noise in communication, it is essential to ensure that each signal is easily recognizable (detectable) and stable (immune to external agents that may distort the signal). These are the two dimensions of the quality of a signal. For the sake of generalization, let us take the example of the written language. The development of typography takes into account letter distinguishability, but it is limited to historical usage of fonts. In terms of readability, the letter “g” is less distinguishable from the letter “q” when compared to the letter “x”, for most typefaces used. Any environmental effect on the printed message (excess or lack of ink in a specific location of the body of the letter, for instance) may result in the misreading of the letter, which is more likely to happen for less distinguishable letters [8] as can be seen in the example illustrated in Figure 2. In his manuscript, Shannon discusses the different levels in which noise can affect a signal, ranging from simple distortion, in which the original signal can still be recovered, to the case where noise makes the signal to be mistaken for another variable.

For the sake of simplicity, and due to the large prevalence in our information technology, we can focus our analysis on the particular case of communication via binary code. The arguments can be generalized to other types of communication.

The issue of signal stability is more fundamentally discussed by Landauer [9]. In his landmark manuscript entitled “*Irreversibility and Heat Generation in the Computing Process*” he focused on the physical foundations of computation. At first, it might seem to be a separate discussion from that of communication but it is essentially the same, once we recognize that information processing requires communication between the processor and memory devices. In other words, computation is intimately correlated to information transmission, storage, detection and recovery, i.e., communication. In his manuscript, Landauer states “*The computing process, where the setting of various elements depends upon the setting of other elements at previous times, is closely akin to a measurement”,* i.e., computing implicitly requires memory and is similar to the engineering problem of measurement.

**Figure 3:**
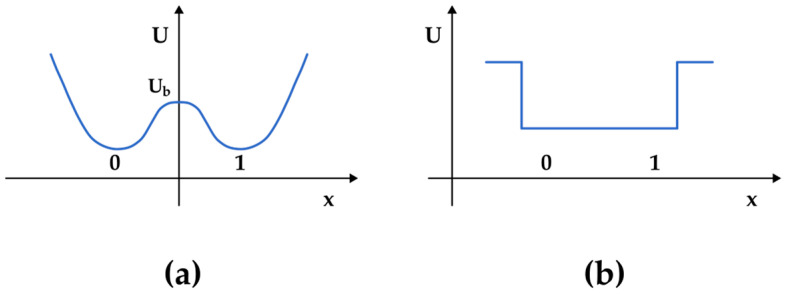
Potential wells sketched by Landauer while discussing dissipation of energy in the computing process. (**a**) magnetic and ferroelectric memories, (**b**) cryotron memory, messages written on a page and a particle inside a box.

The most fundamental aspects discussed in Landauer’s manuscript are the switching process between the states “0” and “1” and the mechanism of preservation of such information for computer processing. Landauer demonstrates that, for the effect of information preservation, the switching mechanism must be dissipative in terms of energy. He analyzes several types of memory devices according to the respective potential well between the states “0” and “1”. For instance, magnetic memories (the kind used in hard disk drives) have a potential well as illustrated in Figure 3a. But there might be cases where the states “0” and “1” are not separated by a potential barrier as in Figure 3b. According to Landauer, this may correspond to a system consisting of a particle inside a box, hinting a connection to the legacy of Szilard’s work. In this case, information is preserved only if the random motion of the particle is slow.

In his manuscript, Landauer devotes great length to discussing a process called “restore to one” which is the equivalent of an “erase” or a “reset” for memory devices. The process is essentially the switching mechanism from the state “0” to the state “1” and, in Landauer’s words “*this energy difference is dissipated and corresponds to the one-half hysteresis loop area energy loss generally associated with switching*”. This is the most important sentence of Landauer’s manuscript. In the case the cycle is completed, i.e., the switching process starts from “0” to “1” and goes back to “0”, the dissipated energy would correspond to the totality of the hysteresis loop area.

It is important to stress that Landauer claims “*The detailed connection between logical irreversibility and entropy remains to be made*”, and later on he states, “*note that our argument here does not necessarily depend upon connections, frequently made in other writings, between entropy and information*”, thus doubting the straightforward connection between information technology and thermodynamics. His manuscript states that the lower bound for this energy dissipation should be kTln2 and this is known nowadays as the “Landauer limit”. However, taking into account that he referenced the book “*Science and Information Technology*” by Brillouin, and referred to the case of a particle in a box as a memory device, strongly indicates that this statement was simply reproduced from Brillouin’s book and Szilard’s manuscript, where this lower limit has already been asserted. Moreover, regarding this lower limit, Landauer declares: “*Naturally the amount of heat generation involved is many orders of magnitude smaller than the heat dissipation in any practically conceivable device*”. This assertion is still valid today [10], and reinforces the disparity, already pointed out by Landauer, between practical logic operations and the assumption of a fundamental link between information and thermodynamics.

### 3.2. Hysteresis Loops as the Cornerstone of the Landauer’s Principle

The term “hysteresis” derives from a Greek word meaning “lagging behind” that already hints something of a “memory”. Scientific studies using hysteresis curves were established for the investigation of magnetic materials in the late XIX century by Alfred Ewing.

A manuscript entitled “*Temperature Gradients as Data Storage Principle*” published by Schoenmaker et al. showed that all memory devices are essentially based on a system that presents a hysteresis loop, in agreement with Landauer’s statement regarding the origin of the fundamental energy dissipation in logic switching. Furthermore, the manuscript puts forward the concept that hysteresis loops are as essential to understanding memory devices as thermodynamic cycles are to heat engines [11]. As stated in the manuscript “*The kinship between hysteresis loops and thermodynamic cycles is multifaceted: their internal area and shape give important information regarding the performance of the device they are associated with and are related to the energy dissipated or produced. (…) Even today, hysteresis loops are primarily regarded as a laboratory characterization technique rather than a theoretical framework. They have become a cornerstone in the characterization and development of magnetic materials*”.

**Figure 4:**
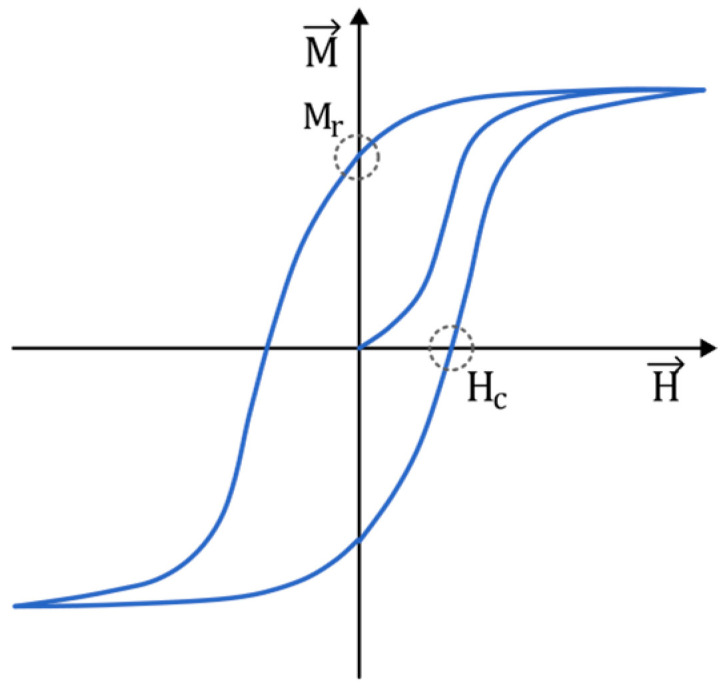
Typical hysteresis loop of a magnetic thin film used in HDD technology. The graph correlates the applied magnetic field (H) and the magnetization of the media (M). An entire cycle corresponds to a transition from a fully magnetized state (magnetization saturation) in one direction, going to a fully magnetized state in the opposite direction and then back to the original saturation state. One-half of the cycle corresponds to the “restore to one” process described by Landauer, as the presence (or not) of a transition between magnetized states defines “1” (or “0”) in this type of device. In order to prevent environmental destabilization of the data, engineers seek to develop magnetic media with high values of coercive magnetic field (H_c_). On the other hand, the larger the remnant magnetization (M_r_), the easier the information is detected. Thus, a large internal area of the hysteresis loop means the information is more detectable and less susceptible to noise, and the energy dissipation in information processing is large.

To illustrate the relevance of hysteresis loops in understanding and characterizing a memory device, we can take the example of a hard disk drive (HDD). For many decades, HDDs have been the main technology for data storage systems and are based on the magnetization state of a section of a magnetic thin film. In the data recording procedure, the “0s” and “1s” states are defined in terms of whether designed areas of the film are magnetized in opposite directions (or not). In the data reading procedure, the head flies over the disk track detecting whether a magnetization reversal occurs for state “1” (or not for state “0”). Figure 4 shows a typical hysteresis loop for a magnetic film used in HDDs depicting the behavior of the magnetization (M) as a function of the applied magnetic field (H). Two main parameters are highlighted. The remnant magnetization (M_r_) is the magnetization state of the media that remains after the saturation, when the applied field is removed. The coercive field (H_c_) is the intensity of the applied field in the opposite direction necessary to bring the magnetization to zero after the media has been saturated. A high H_c_ means that the magnetization state is less susceptible to demagnetization effects by thermal fluctuations and other environmental factors. More specifically, the magnetic anisotropies of the media are engineered in the way that there is a high potential barrier (U_b_ in Figure 3a) between the two directions of magnetization. A high M_r_ means that the bit is more easily detected. High H_c_ and M_r_ yield better signal-to-noise ratio, making the information more stable and measurable, which is the core of the discussion in “*A Mathematical Theory of Communication*” by Shannon.

Another remarkable property of hysteresis loops, already mentioned by Landauer, is that it is possible to demonstrate that its internal area is proportional to the energy dissipated during the cycle.

In the magnetic case, the calculation follows the expression:

(2) $$\frac{Work}{Volume}=\int_{0}^{M_{final}}HdM$$

Thus, the larger the internal area, the better the quality of the information stored, and bigger is the amount of energy dissipated in the respective computing process. This is the practical connection between information and thermodynamics stated by Landauer, which portrays a distinct view from the ones put forward by Brillouin and Szilard.

### 3.3. How Statistical Mechanics Explains Classical Thermodynamics

Before we discuss the works of Szilard and Brillouin, it is necessary to draw attention to settled matters in statistical mechanics. This section may appear to be superfluous, but we maintain that it is necessary in order to provide a solid foundation for underpinning our examination of some of the arguments put forward by Szilard, Brillouin, Bekenstein, and Hawking. We depart from the established fact that statistical mechanics is a very successful field of research in physics. Currently used in several different areas, it started as a way to explain the main aspects of classical thermodynamics.

For historical and practical reasons, work extraction by means of the expansion of a gas in a piston–cylinder system is a classical problem in thermodynamics. Statistical mechanics was developed afterwards in order to give physical meaning to thermodynamic parameters such as pressure and temperature, justifying them as the result of the statistical behavior of very large numbers of particles, typically on the order of 10^24^. Szilard’s 1964 manuscript is a departure from the piston–cylinder construction [7].

**Figure 5:**
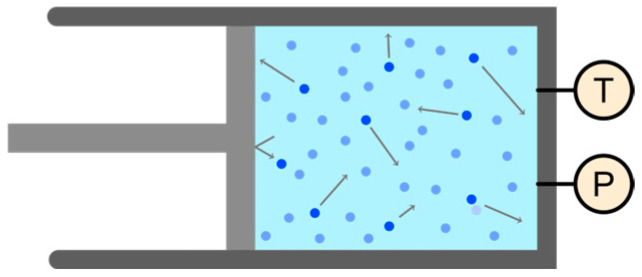
The gas inside a piston–cylinder arrangement is the most classical thermodynamic system since it was studied in order to understand steam-engines. Statistical mechanics discussions within this framework gave rise to the Maxwell demon problem and Szilard engine.

Consider a piston–cylinder system equipped with a thermometer and a pressure gauge as depicted in Figure 5. In the following, we reproduce how Feynman describes the pressure and temperature of a gas in such a system [1] (p. 1–3): “*Let us see what some of the properties of steam vapor or any other gas are. The molecules, being separated from one another, will bounce against the walls. Imagine a room with a number of tennis balls (a hundred or so) bouncing around in perpetual motion. When they bombard the wall, this pushes the wall away. (Of course we would have to push the wall back.) This means that the gas exerts a jittery force which our coarse senses (not being ourselves magnified a billion times) feel only as an average push. In order to confine a gas we must apply a pressure. (…) If we increase the temperature without changing the density of the gas,* i.e.*, if we increase the speed of the atoms, what is going to happen to the pressure? Well, the atoms hit harder because they are moving faster, and in addition they hit more often, so the pressure increases. You see how simple the ideas of atomic theory are*”. Feynman also describes the relation between work and the parameters of temperature and pressure: “*Suppose that the piston moves inward, so that the atoms are slowly compressed into a smaller space. What happens when an atom hits the moving piston? Evidently it picks up speed from the collision. You can try it by bouncing a ping-pong ball from a forward-moving paddle, for example, and you will find that it comes off with more speed than that with which it struck. (Special example: if an atom happens to be standing still and the piston hits it, it will certainly move.) So the atoms are “hotter” when they come away from the piston than they were before they struck it. Therefore all the atoms which are in the vessel will have picked up speed. This means that when we compress a gas slowly, the temperature of the gas increases. So, under slow compression, a gas will increase in temperature, and under slow expansion it will decrease in temperature*”. Thus, it is understood that temperature and pressure are the result of the statistical giggling of ~10^24^ particles and that a very large number of particle–wall interactions are involved in the process of work submitted to or extracted from a gas.

Note that if we want to extract work *from* the system, it is the pressure of the gas that *drives* the piston during expansion. On the contrary, if we want to *act* on the gas, like in a compressor, then we have the presence of an *external force* acting on the piston. This aspect is going to be noteworthy in our analysis of the Szilard engine.

However, how does statistical mechanics describe the properties of an ideal gas inside a piston–cylinder system? To illustrate this point, we calculate the volume occupied by a gas in the phase space (the partition function Ω which accounts for the number of states of the system) following the procedure found in a reference textbook on the subject written by F. Reif [12]. This treatment is performed considering the microcanonical ensemble and the Cartesian coordinate system. Consider a gas of N particles of mass m inside a container of volume V. The energy of the system is given by:

(3) E=K+U,

where K is the kinetic energy and U is the potential representing the interactions between the particles of the gas. The phase space is composed of the momentum (p) and position (q) of each particle in the system. One can say that the volume occupied by the system in the phase space is the product of the volume occupied by the possible positions of the particles (φ) multiplied by the volume occupied by the possible momenta of the particles (χ). Thus, the partition function is of the form:

(4) $$\Omega \left(K,U\right)=\varphi \left(q\right)\chi \left(p\right).$$

Evaluating K by decomposing the momentum p in the Cartesian reference we have:

(5) $$\sum_{j=1}^{N}\sum_{\alpha =1}^{3}\frac{p_{\alpha j}^{2}}{2m}=K$$

where summation in j represents the totality of the particles of the system and the summation in α represents the coordinates x, y and z. Equation (5) is the expression for a hyper-sphere of radius:

(6) $$p=\sqrt{2mK}$$

Thus, χ is proportional to the volume of a 3N-dimentional spherical shell of radius p, hence:

(7) $$\chi \left(p\right)={\left(2mK\right)}^{\frac{\left(3N-1\right)}{2}}\approx {\left(2mK\right)}^{\frac{3N}{2}},$$

where 1 is neglected when compared to N, which is naturally very large.

To assess φ we have to evaluate the possible positions presented by the particles of the system. To accomplish this, we have to take into consideration the influence of U. However, if one considers a gas with rather small density and rather high kinetic energy (as an ideal gas at room temperature), one can consider U as negligible [12]. Thus, each particle contributes to *φ* with the volume of the container V:

(8) $$\varphi \left(q\right)=V^{N},$$

and Ω is given by:

(9) $$\Omega \left(K,U\right)=BV^{N}{\left(2mK\right)}^{\frac{3N}{2}},$$

where B is a constant to guarantee proper proportionality. By applying the Boltzmann-Planck equation:

(10) S=klnΩ,

we arrive to the expression for the entropy of the gas:

(11) $$S=kN\ln B+\;kN\ln V+kN\frac{3}{2}\ln\left(2mK\right).$$

To bring this development closer to a more familiar setting, one can apply the known thermodynamic expression:

(12) $$P=kT\;\frac{\partial ln(\Omega )}{\partial V}$$

to Equation (9) and one finds the ideal gas equation:

(13) PV=NkT

where k is the Boltzmann constant and T is the temperature of the gas.

It is important to note how, in the line of reasoning about φ, the potential U is disregarded and the parameter V is introduced with the plain argument that the allowed room for each particle of the gas is the volume of the container. At this point, Reif inserts a footnote in his book stating that this could be done by making the potential energy of a particle go to infinity once it tries to penetrate the wall of the container [12] (p. 64). The reason this is stated in a footnote is that the physical basis of the argument is poor. In his work, Szilard discussed how an intelligent being is able to “know” the position and velocity of a gas particle, and use this information in order to influence the system. Conversely, how could a particle “know” that it is contained by a box of volume V if the walls are not physically modeled? As we are going to see, containers are often poorly defined in statistical mechanics, and this is crucial for the subject in this manuscript.

Another relevant aspect is that the classical statistical model of an ideal gas considers that the particles follow the Maxwell–Boltzmann distribution, and this is achieved by treating the collision between particles in a special way: instead of following classical mechanics, interaction between particles is subjected to the H-theorem, which assumes that in each collision, the kinetic energy of the particles is assigned according to a statistical probability.

**Figure 6:**
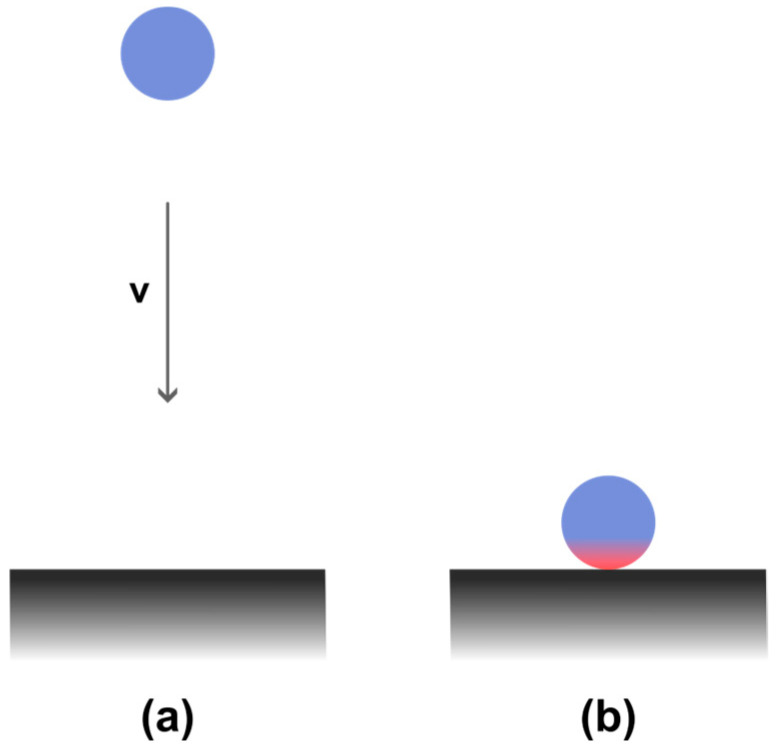
Different manifestations of kinetic energy. (**a**) A steel ball is free-falling. In this case, the kinetic energy is manifested as a mechanical phenomenon that can be modeled as a single body with velocity v. Essentially, all atoms of the ball move coherently. (**b**) At the moment of impact with the floor, the energy is dissipated as heat, sound vibrations and material deformation. Now, the manifestation of kinetic energy is essentially in the form of heat and has to be modeled statistically. This example also indicates the relevance of the interaction of the particles of a statistical mechanical system with walls (the floor in this case). This issue is essential for understanding the implications of the Szilard engine.

Furthermore, as we saw in the explanation by Feynman, physicists explain temperature as a manifestation of the kinetic energy of the particles of the gas. In this sense, it is salutary to discuss this issue deeper. Not all manifestations of kinetic energy are related to heat. Let us analyze the energy transformations in the example case of a steel ball with mass (say m=100 g) falling from a height (h=50 m a ~15-floor building) as illustrated in Figure 6. Initially, the ball has gravitational potential energy that is gradually transformed into kinetic energy as the ball descends. This kinetic energy is not manifested as heat until it reaches impact with the floor. During the descending, the problem is essentially described by the physics of classical mechanics and the ball can be modeled as a body positioned in a height h with velocity v. The problem is not statistical because all the atoms that constitute the ball are moving coherently (of course, if the temperature is above absolute zero, the atoms are giggling accordingly, but this is secondary to the analysis in this situation). The conditions change only at the moment the ball reaches the ground, when the kinetic energy is dissipated in the form of heat, sound, vibrations and material deformation during impact.

Some calculations using conservation of energy where g is gravity’s acceleration, and c is the specific heat capacity of steel, we have:

(14) $$mgh=m\frac{v^{2}}{2}=mc∆T,$$

from where we may estimate that the temperature of the bottom part of the ball may rise by about 1 K. As it is clear, the ball only “sensed” about the floor at the event of impact, when there was interaction between the electromagnetic potentials of the ball and the surface. At this point, the kinetic energy ceased to be expressed as coherent motion and turned to a thermal manifestation, where its modeling can only be achieved by statistical mechanics.

### 3.4. Szilard and Brillouin

To put it shortly, the work of Brillouin on information physics was strongly influenced by the works of Szilard, which in turn was influenced by the “Maxwell’s demon” problem, a thought experiment that is the source of unsettled debates. We argue that, essentially, this process is the source of unsound assumptions that led to the black hole information paradox.

In 1867, J. C. Maxwell proposed a thought experiment where a “finite being” (that is now called Maxwell’s demon) could control a door between two gas-filled chambers, acting in a molecular level by sorting between fast and slow particles in order to violate the Second Law of thermodynamics. Ever since, a great deal of research has been done on this matter [13].

In a first level of analysis, it is curious that an entire research program would be committed to discuss and experiment on a physical problem involving a supernatural being [14]. A good part of this research program has been devoted to debate and characterizing how this demon could be manifested by physical systems: how could the demon detect the position and velocity of individual molecules (demon’s eyes)? How could the demon actuate on a door (demon’s arms)? And more interestingly, how could the demon process the information for such activity, or in other words, how does the demon “know” or “understand” his surroundings? And this last question brings the connection between Maxwell’s demon and the black hole paradox. It is not surprising that many experiments demonstrated how the Maxwell demon is “exorcised”, i.e., that no violation of the Second Law could be achieved [10]. Interestingly, as the experimental demonstrations make use of sensors, processors, feedback control, and actuators, the situation falls back to the framework discussed by Shannon and Landauer, related to signal-to-noise ratio in communication and switching energy dissipation in computation, i.e., the solution befalls on the solid works of Shannon and Landauer, and the Maxwell demon is exorcized by a process akin to a measurement performed by a receiver.

**Figure 7:**
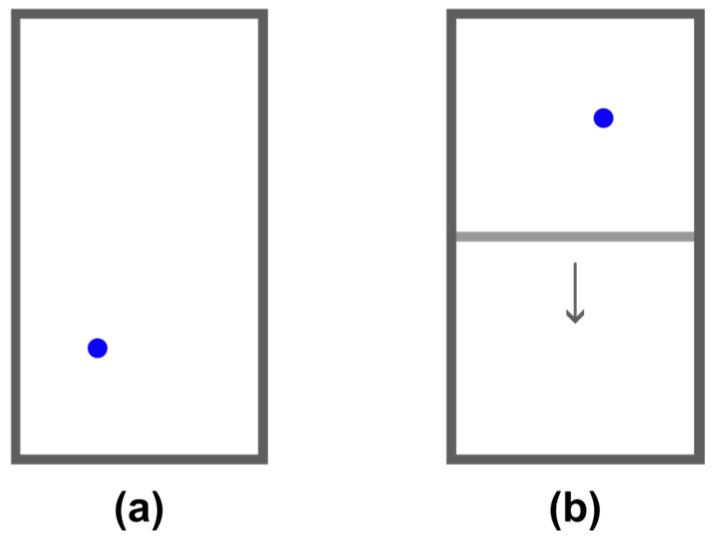
The Szilard piston–cylinder system. (**a**) A single molecule gas is trapped inside a cylinder closed at both ends. (**b**) By knowing that the molecule is located in the upper part of the cylinder, an intelligent being introduces a piston in the middle and moves it downward.

The 1964 Szilard’s manuscript entitled “*On the Decrease of Entropy in a Thermodynamic System by the Intervention of Intelligent Beings*” deals with a particular formulation of Maxwell’s demon problem. The adjective “intelligent” stands out, indicating that the being must “understand” its surroundings. Szilard discusses at length about the nature of this intelligent being. He states that it could not be biological as a “*nervous system is dependent on continual dissipation of energy*”. Szilard conceptualizes “*a sort of deus ex machina, one who is continuously and exactly informed of the existing state of nature and who is able to start or interrupt the macroscopic course of nature at any moment without expenditure of work*”. The aim of his manuscript was to investigate the conditions which allow the construction of a perpetual motion machine of a second kind. This on itself is a debatable purpose, with strong influence of Maxwell’s demon thought experiment. However, we would like to focus on Szilard’s thought experiments. In the most acknowledgeable of its variations, he considers a cylinder closed in both ends that encapsulates a gas consisting of a single particle (Figure 7). By knowing that, for instance, the particle is in the upper part of the cylinder, the intelligent being could insert a piston and extract useful work. As Szilard puts it “*then he would move the piston slowly downward until it reaches the bottom of the cylinder. During this slow movement of the piston the molecule stays, of course, above the piston. However, it is no longer constrained to the upper part of the cylinder but bounces many times against the piston which is already moving in the lower part of the cylinder. In this way the molecule does a certain amount of work on the piston. This is the work that corresponds to the isothermal expansion of an ideal gas*”.

According to Szilard, this process could be repeated by the intelligent being indefinitely, thus violating the Second Law.

We argue that, beyond relying on the framework of Maxwell’s demon problem, there are several other issues with Szilard’s work that undermine his main conclusions. We perform here *a reductio ad absurdum* in several levels.

First, he conceived a statistical mechanical system consisting of a single particle. As we saw in previous sections, pressure and temperature are statistical manifestations of many, many particles (typically on the order of 10^24^). A detector could measure the energy of a single particle, but a thermometer could never measure the temperature of it. When discussing Figure 6, we saw that kinetic energy can manifest in different forms. What is the temperature of a single particle? How would the H-theorem work for this gas? Would the particle, in each interaction with the thermalized wall, acquire a different kinetic energy? Would that mean that each interaction would result in a gas with a different temperature? Many could argue that this issue could be circumvented by the ergodic principle. However, it can be demonstrated that thermalization is more related to large degrees of freedom than to ergodicity [15], and that, while for a realistic gas the equilibration time scale is a fraction of a second [16], for a single particle gas this scale is arguably ~10^24^ times longer, making the Szilard engine unrealistic even as a model. From here we may follow a series of paradoxes that arise from inconsistent assumptions. If a system is to follow classical thermodynamics laws, it has to be constituted by a very large number of degrees of freedom. On the other hand, there are several works exploring the limits of systems with restricted degrees of freedom that violate accepted limits of thermodynamics such as Carnot efficiency [17], and the Second Law [18]. Works on the realization of Maxwell’s demon and Szilard engines should be analyzed taking this into account [19,20,21].

Second, from previous sections, it is established that if we want to extract work from the system, it is the pressure of the gas that moves the piston. What is the pressure of a single particle gas interacting with the piston wall? How is this interaction modeled? Can pressure be modeled as a succession of single collisions? How is the piston modeled? Is it composed of a single particle, or by ~10^24^ particles? As a matter of fact, a single molecule gas could hardly move any real piston due to reasons usually raised within the Maxwell’s demon research program. Furthermore, there is another problem with the work extraction process devised by Szilard. In his words, the being “*would move the piston slowly downward until it reaches the bottom of the cylinder*”. If the intelligent being is the driving force that moves the piston, no work is extracted from the system. Szilard explains this matter further when he states “*The man moves the piston up or down depending on whether the molecule is trapped in the upper or lower half of the piston. In more detail, this motion may be caused by a weight, that is to be raised, through a mechanism that transmits the force from the piston to the weight, in such a way that the latter is always displaced upwards. In this way the potential energy of the weight certainly increases constantly. (The transmission of force to the weight is best arranged so that the force exerted by the weight on the piston at any position of the latter equals the average pressure of the gas.) It is clear that in this manner energy is constantly gained at the expense of heat, insofar as the biological phenomena of the intervening man are ignored in the calculation*”. This reinforces that Szilard misunderstands how work is extracted from piston–cylinder engines. He states again that “the man moves the piston up and down” or that the force that the man exerts could be replaced by a weight. How is it possible that the movement of the piston “may be caused by a weight” at the same time that the extracted work is manifested by the upward movement of the same weight?

Third, there is an even more fundamental problem with Szilard’s thought experiment. Note that Szilard keeps referring to the single molecule as a “gas” that exerts “pressure”. So let us assume that this single particle inside a piston–cylinder system, envisaged by Szilard, behaves like a gas as described by Feynman. Before the intelligent being introduces the piston (Figure 7a), the gas fills the total volume of the cylinder $V_{0}$. At the moment the being inserts the piston, the gas is confined in half of its initial volume, and we call this new volume $V_{1}$. This would represent an instantaneous decrease in entropy given by:

(15) $$∆S=kN\ln\left(V_{0}-V_{1}\right)=k\ln\left(V_{0}-V_{1}\right)$$

where N=1, as it is a single molecule gas. This entropy variation should be compensated by a temperature increase in the gas. An analogous procedure for a real piston–cylinder system would be a compression of the gas by the piston until the volume reaches half of its initial volume. In this scenario, during the compression, the particles would interact with a wall that moves into the bulk of the gas, increasing the kinetic energy of the particles with each interaction, thus increasing the temperature of the gas as described by Feynman in previous sections. Therefore, the increase in temperature is justified by the interaction of the particles with the moving wall of the piston. In the case of Szilard experiment, the piston is positioned in the middle of the cylinder with no interaction with the molecule. How would the particle “sense” that the volume of its container has decreased? This could, presumably, only happen when the particle interacts with the piston. Note that, as the cylinder could be arbitrarily large and the kinetic energy of the particle arbitrarily low, the period of “unawareness” of the particle could last for an arbitrarily long time. Only the intelligent being “knows” that the particle is suddenly confined in a much smaller volume. The particle is “unaware” of the introduction of the piston for an arbitrarily long time. Note that in the case of a real gas, there is no meaning in saying that the gas is “unaware” because it was compressed by the piston, or in other words, it “sensed” the work performed by the pressure of the piston by means of a very large number of interactions with the walls.

Furthermore, and lastly, the problem goes even deeper. The 1964 Szilard manuscript is considered by many the first work to connect information to statistical mechanics. According to Szilard, the intelligent being must know if the particle is up or down in order to decide regarding the movement of the piston. Szilard understands that the work is extracted by the expansion of the gas; however, in his argument, the being must know where the particle is located (must have the information) in order to move the piston in the right direction. However, as happens for real piston–cylinder engines, it is the pressure of the gas that moves the piston, so the work would be extracted regardless of the position of the particle. If the particle is up, its pressure would push the piston down, and vice versa. In other words:

**Remark 1.**  *If we accept that a single molecule system would behave like a real gas, the work could be extracted regardless of the position of the particle and the decrease in entropy could be achieved by an “unintelligent being”. This undermines the connection between information and thermodynamics put forward by Szilard’s work.*

Thus, expressing in a concise way and allowing a degree of language abuse, we can say that the Szilard piston–cylinder engine works, not because the intelligent being knows where the particle is, but because the particle “senses” the presence of the piston (inserted by an unintelligent being) and pushes the system into expansion by exerting pressure (a statistical mechanical manifestation of a gas). There is no actual information involved once we consider that a particle is not able to “know” anything.

Despite all the shortcomings, interestingly, Szilard reasons that, at the end, the Second Law would not be violated, once it is recognized that the intelligent being, in order to know the position of the particle, should make a measurement and this process requires memory, in accordance with our previous discussion. As he states “*A system in which such measurements occur shows a sort of memory faculty, in the sense that one can recognize by the state parameter y what value another state parameter had at an earlier moment, z and we shall see that simply because of such a memory the Second Law would be violated, if the measurement could take place without compensation*”. And then he follows by saying that such measurement would be accompanied by a production of kln2 units of entropy. This is stated at least 32 years before Landauer publishes his landmark paper. Szilard dedicates a good portion of his manuscript justifying the minimum entropy production and makes use of an Ansatz in order to arrive at the value of kln2. As far as the authors of this manuscript are concerned, the Landauer limit should be called the Szilard limit.

In 1956, Leon Brillouin published the book entitled “*Science and Information Theory*” [6], that sought to establish a direct fundamental connection between information and thermodynamic entropies. This would allow the usage of Shannon’s formula for general problems in physics, with the black hole entropy as a notorious example.

Brillouin’s book is heavily based on the work of Szilard, which is referenced repeatedly throughout the book. Brillouin dedicated an entire chapter to Maxwell’s demon and the connection between entropy and information. In this chapter, he states “*Szilard published in 1964 a very remarkable paper on the problem of Maxwell’s demon and discovered for the first time the connection between information and entropy*”. Note that Brillouin uses the term “discovered” instead of “proposed”. This is a questionable stance in terms of philosophy of science, especially considering that we just subjected Szilard’s conclusions to a *reductio ad absurdum*. In his analysis of the Maxwell’s demon, Brillouin commits all the inconsistencies practiced by Szilard: a gas constituted by a single particle, the position of the particle is discovered by the observer (this is how he calls the “intelligent being”) and the observer is the actor that moves the piston, etc. He is even more explicit in equating a single particle to a classical gas by stating “*the whole system is maintained at a constant temperature T by a thermostat. The molecule will collide with the piston many times during such operation, and these collisions result in an average pressure similar to the one of an ideal gas*”.

Interestingly, Brillouin states that Shannon rediscovered the connection between entropy and information, despite the fact that Shannon never supported such a direct connection. We note that there is a transition from something believed to be similar to something believed to be directly interchangeable physically without further justification, even looking backwards in reference works. As we are going to see, a similar pattern happened in black hole thermodynamics.

Another aspect worth noticing is that, by constantly citing Szilard, the lower bound entropy production kln2 is used in Brillouin’s analysis frequently. Note that Brillouin published his book 5 years before Landauer published his manuscript.

In order to put forward his ideas, Brillouin departs from the definition of entropy given by Shannon’s formula, adjusting it to a specific proportionality, by adding the Boltzmann constant k, turning Equation (1) identical to the known Gibbs entropy formula. He then defines a distinction between free and bound information as follows

- “*Free information* $I_{f}$ *occurs when the possible cases are regarded as abstract and have no specified physical significance*”;
- “*Bound information* $I_{b}$ *occurs when the possible cases can be interpreted as complexions of a physical system. Bound information is thus a special case of free information*”.

As we are going to see, these definitions are problematic, especially because Brillouin goes on to explain that “*only information connected with certain specific physical problems, that is bound information, will be thought of as related to entropy, (…) In the case of free information, we prefer not to think of a connection between information and entropy*”. At the time, he explains the concept of bound information, he states the term “complexions”, used by Planck, that might be transformed in modern jargon into what physicists nowadays call “internal configurations” or “quantum states”. Note that the definition of free information is quite vague and it does not specify if it is related to complexions or not.

Brillouin goes on to define a generalized Second Law (that he calls “Carnot’s principle”) related to a process that brings a physical system from an initial state “0” to a final state “1” with the form:

(16) $$∆S_{1}\geq 0\;\;,\;\;\;\;\;or\;\;\;\;\;\;∆\left(S_{0}-I_{b}\right)\geq 0.$$

According to Brillouin, $-∆I_{b}$ must be supplied by an “external agent” whose entropy increases. From this, he concludes bound information $I_{b}$ decreases entropy S, and is defined as negentropy N.

As Brillouin sought to connect the works of Szilard to the work of Shannon, he contextualized his definitions of free and bound information into the context of a person communicating a message to a friend. He divides this process into five different steps that can be correlated to the steps devised by Shannon, as illustrated in Figure 1. In his words: “*We start with an individual in possession of information, which will be free as long as it remains in his mind and is, thus, not directly connected with any physical system. We follow the losses in this information as it is transmitted to a second individual, and at each step we state whether the loss is of free or of bound information:*

*A.*  *An individual possesses information which is free;*
*B.*  *He tells a friend about it in English, say, and the information is now bound: it has been transformed into sound waves, or electric pulses, or some other physical disturbance which may be used for communication. There may be errors in the coding for transformation. This will be a loss of free information, since it occurs before the transformation into a physical disturbance;*
*C.*  *Due to distortion and thermal noise in the communication system, some of the information may be lost. This is loss of bound information;*
*D.*  *The friend is hard of hearing, so that he misses a few words. The loss here is of bound information, but the information received by the friend is now free since it is in his mind;*
*E.*  *After a while the friend forgets some of the information, and this loss, too, is a loss of free information”.*

Three aspects we would like to stress regarding this description.

First, Brillouin equates the information in the mind of a person to free information, the kind that he defines as not connected with any physical system. Moreover, he states that free information can be lost as well, in different steps of the process. We may ask: when free information is lost, it turns into what? Is free information consisting of quantum states (complexions)? For instance, when the individual speaks to his friend and there are errors in the coding for transformation (he mispronounces a word) are not the soundwaves produced composed of complexions? For the sake of context, in the introduction of his book, Brillouin (probably influenced by Shannon) declared that “*our definition of information is an absolute objective definition, independent of the observer. (…) All these elements of human value are ignored by the present theory*”. On the other hand, his work is strongly based on Szilard ideas regarding the actions of an “informed being” on a physical system, justifying the connection between information and thermodynamics. This is reflected in his definition of bound information, that is “negentropy supplied by an external agent”. It is also not explained what happens to a bound information when it is lost. This is so elusively defined that it sounds contradictory. This is even more evident later on, when Brillouin states, “*It should be noted that the losses in steps B, D, and E are borderline cases. Sometimes it is difficult to distinguish between free and bound information, particularly in cases involving a human observer. We must, of course, ignore any human value. For example, we exclude the case of a scientist collecting data and then discovering a scientific law as a result of study of the data. Our definitions do not apply to such a problem. It is only by excluding the process of thought that we have been able to develop a satisfactory theory of information*”.

Second, we understand that a noisy signal, that generates information loss, still dissipates energy in a communication process in any real system, and contributes to entropy as well. If we go along with Brillouin’s description, at the time a bound information is lost, we may ask whether the distorted signal is or not related to quantum states (complexions). This is also problematic. Take for instance, the effect of noise in the case of Figure 2 when the word-extracting software worked on the word “statistical”. Part of the information was lost due to noise, but the entropy generated by that processing was still equivalent. How different it was, energetically, for the computation process, to identify the noisily deformed “c” letter as a “r”? But according to Brillouin definition, the bound information lost in the process did not enter into the calculation expressed by Equation (16).

Third, Brillouin emphasizes in step C the role of noise, but does not clearly define a distinction between signal and noise.

The book offers two physical problems in order to demonstrate how bound information arises and its thermodynamic role. The first problem is the case of a gas contained in a volume $V_{0}$ and afterwards the gas is allowed to expand into a larger volume $V_{1}$. To analyze this problem Brillouin used the Sackur–Tetrode equation for the entropy of an ideal gas:

(17) $$S=kN\left[\frac{5}{2}+\ln\left(g\frac{V}{N}{\left\{\frac{4\pi mE}{3h^{2}N}\right\}}^{3/2}\right)\right]$$

where h is the Plank constant, N is the number of atoms in the gas, m is the mass of the atom, E is the total energy, and g is the number of indistinguishable states on the ground level of the atom. Equation (17) represents a refinement from Equation (11) using tools from information theory. As we are going to see, a detailed understanding of Equation (17) is not necessary for the purpose of our analysis, that emphasizes two aspects. First, g is a parameter that designates a quantum state. Brillouin asserts that different values of g does not generate a measurable discrimination in any physical manifestation of the gas, therefore quantum states related to g could not correspond to bound information. Thus, by this example, we have the confirmation that not every quantum state (or internal configuration of a system) corresponds to information. The second aspect is related to how the volume V is introduced in Equation (17). This happens in a similar manner as in Equation (11), by stating that each particle has access to a volume V in the phase space. In information science, this is equivalent to saying that each particle “knows” that their position is restricted to a volume V. With this reasoning, when the gas is allowed to expand from the initial volume $V_{0}$ to a larger volume $V_{1}$, there is a variation in entropy that corresponds to a variation in information given by:

(18) $$\Delta S=S_{1}-S_{0}=kN(\ln V_{1}-\ln V_{0})=\Delta I.$$

In which Brillouin follows to explain that “*the gas progressively forgets the information*” during the expansion process, again attributing to the particles the ability of “knowing” about their environment. Curiously, a footnote is inserted along with this statement that declares: “*J. von Neumann also considers the case of the observer forgetting the information, and states that this process also means an increase of entropy. Here, again, we see the impossibility of always distinguishing clearly between free and bound information*”. So, here it seems that information science is in a paradoxical situation that we call the information bearer paradox:

**Remark 2.**  *The information bearer paradox of the Brillouin’s expanding gas example: On one side, the faculty of “knowing the information” is attributed to the atoms in the gas, which is highly questionable. On the other hand, the faculty of knowing is attributed to the observer, which is also questionable in a field of research that claims that all elements of human value are ignored and that information in the mind of an individual is free information, the kind that cannot be associated with negentropy. Note that the physical behavior of the gas would not change in the case where the observer was not in the room or had no knowledge of the process being taken place.*

This paradoxical situation happens because Brillouin is trying to generalize Shannon theory of communication to a physical problem of gas expansion. In this situation, there is no “destination for the message” as in the sketch of Figure 1, thus remaining the question “who owns the information?”. There is no message being transmitted from a source to a destination in the expansion of a gas. There is no measurement being made. Despite the effort to generalize Shannon’s theory to physical systems, this represents a wide departure from the scope of Shannon’s work.

It is worth noticing that the parameter V is introduced in Equation (8) using the same justification, however in a pragmatic way, indicating that containers are often poorly modeled in statistical mechanics. Particles cannot “know” that they are confined in a volume V. Particles only interact with energy potentials generated by the walls of the container and other particles. We are going to discuss this issue further in the next section.

The second problem presented by Brillouin, that exemplified how information is directly connected to entropy, is gas diffusion. Again, this example is subjected to the very same flaws expressed for the first example.

As one last important aspect of discussion, in chapter 14 of his book, Brillouin recognizes that in order to acquire information, the process of measurement is necessary. This is another contradiction present in his book, once his gas expansion example does not involve a measurement. Then, he calculates the minimum energy necessary for measurements in different typical laboratory experiments. In each calculation, a factor α≫1 is included. As he explains, “α *is assumed to be large enough in order to reduce errors of observation and detrimental operations of the system. This factor* α *ensures reliability in the experimental device*”. Therefore, he recognizes that, for the sake of signal-to-noise ratio, the energy required for any practical measurement has to be significantly higher than the calculated minimum.

### 3.5. Partial Conclusions

Before we discuss the process that resulted in the establishment of black hole thermodynamics, it is salutary to stress the main conclusions of this section.

Shannon devised a consistent mathematical theory of communication. He did not believe in the direct connection between information and thermodynamic entropies. His main motivation was the mathematical understanding of the role of noise in communication. In his description of the process, the role of the receiver is evidenced. The existence of a receiver implies a measurement process.

According to Landauer, every measurement, as well as any act of computing, requires memory and the process of switching. He states that the energy consumed in the computing process is proportional to the internal area of the memory-unit hysteresis loop. This energy is essentially dissipative. We argue that he asserted the lower bound kTln2 based on previous literature. He believed that any practical switching in computation would require energies orders of magnitudes above this limit.

We demonstrated that a hysteresis loop with a large area is related to information (data) readability and stability. These are essential parameters to engineer signal-to-noise ratio in communication systems.

We subjected the 1964 Szilard’s manuscript to a *reductio ad absurdum*, demonstrating that work could be extracted from his idealized system by an “uninformed being”. Thus, the work of Szilard could not represent a direct connection between information and thermodynamic entropy.

Brillouin’s book did not succeed in generalizing the connection between information and thermodynamic entropies in physical sciences. Many weak points have been highlighted. His work is based on the distinction between free and bound information at the same time it fails to effectively distinguish one from another. Brillouin recognized this failure in his book but believed it could be resolved in the future. When he tried to exemplify his general theory with the problem of the gas expansion, his theory assumed that the atoms “know” they are confined in a container of volume V, which leads to the paradox of the information bearer as there is no measurement involved. What is clear from his work is that not every quantum state is information. Moreover, and paradoxically, he recognizes that information requires a measurement process. When he defines a minimum energy for measurements, he includes a factor α to account for signal-to-noise ratio, thus acknowledging that information processing requires energy levels remarkably above the calculated minimum. This means that information consists of a collection of quantum states. In practice, the size of this collection is arbitrary.

## 4. Black Hole Thermodynamics?

### 4.1. Temperature as an Analogy

In the previous section, we maintained that Shannon considered information entropy as an analogy to thermodynamic entropy. Afterwards, other authors considered them as identical, even referring to Shannon’s work as a proof. In black hole thermodynamics, a similar process happened. Let us analyze the works that served as base for the 1974 Hawking’s manuscript entitled “*Black hole explosions?*”.

In the beginning of his manuscript Hawking states “*it seems that any black hole will create and emit particles such as neutrinos or photons at just the rate that one would expect if the black hole was a body with a temperature of* (κ/2π)(ℏ/2k) *where* κ *is the surface gravity of the black hole*”. In order to justify this statement, he refers to a manuscript published by him, along with J. M. Bardeen and B. Carter. [3]. In the last paragraph he expresses “*Bekenstein suggested on thermodynamic grounds that some multiple ok κ should be regarded as the temperature of a black hole. He did not, however, suggest that a black hole could emit particles as well as absorb them. For this reason Bardeen, Carter and I considered that the thermodynamical similarity between κ and temperature was only an analogy. The present result seems to indicate, however, that there may be more to it than this. Of course this calculation ignores the back reaction of the particles on the metric, and quantum fluctuations on the metric*”. Thus, Hawking is aware that his manuscript represents a transition from temperature as an analogy to temperature as a direct manifestation of thermodynamic temperature. However, in the beginning, he states the temperature as a fact to justify his calculation on the radiation. At the end, he relies on his calculation to explain the temperature.

Let us analyze the referred manuscript that Hawking published with Bardeen and Carter, which is entitled “*The Four Laws of Black Hole Mechanics*”. Note that the term used was “mechanics” instead of “thermodynamics”. This work deals with analogies of entropy and temperature of black holes. At one point, the manuscript states “*It can be seen that* (κ)⁄(8π) *is analogous to temperature in the same way that* A *is analogous to entropy. It should be emphasized that* (κ)⁄(8π) *and* A *are distinct from the temperature and entropy of the black hole. In fact the effective temperature of a black hole is absolute zero*”.

The 1973 Bekenstein’s manuscript is the main reference on this matter [4]. In it, there is a section that exposes the influence of communication technology on black hole thermodynamics. The first sentence of section III he states “*The connection between entropy and information is well known*”. And he cites Shannon’s manuscript and Brillouin’s book [5,6]. Bekenstein departs directly from Shannon’s formula for information entropy, Equation (1). In the following, he expressed the Brillouin connection between information and entropy with the expression:

(19) ΔI=−ΔS,

arguing that Equation (19) is applicable “*to such diverse systems as a quantity of gas in a box or a telegram*”. We demonstrated that this statement is very debatable. Moreover, note that Brillouin went to a great length in order to avoid expressing the relationship between information and entropy in the form of Equation (19) because he was aware of its fragility, especially with the issue of the distinction between bound and free information. On the other hand, Bekenstein did not have such wariness. Afterwards, Bekenstein gives further justifications based on the problems regarding Maxwell’s demons, that we also demonstrated to be based on feeble premises. And without citing Landauer, he states that the minimum amount of entropy increase is associated with a single bit related to a yes-or-no question with value ln2. As he is using Shannon’s formula directly, his entropy evaluation does not have units. Thus, it is clear that he is basing his analysis completely on information entropy, as he states “*In the context of information a black hole is very much like a thermodynamics system*”.

Using arguments of analogy Bekenstein suggests that the entropy of a black hole could be expressed as proportional to its rationalized area σ:

(20) $$S_{bh}=\gamma \sigma .$$

Regarding the constant of proportionality γ he states “*Comparison shows that* γ *must have units of (length)^−^*^2^. *But there is no constant with such units in classical general relativity. If in desperation we appeal to quantum physics we find only one truly universal constant with the correct units:* $\hbar^{-1}$*, that is, the reciprocal of the Planck length squared*”. Note the term “desperation”.

In Brillouin’s vocabulary, information is associated with “complexions” whereas Bekenstein speaks about “internal configurations” that he equates to accessible quantum states of a system. As he argues, a black hole is formed from a very large physical system with a large number of internal configurations and the only information that remains of the system is the black hole’s mass M, charge Q and angular momentum $\vec{L}$, that is analogous to a statistical mechanical system being determined by its volume V, temperature T, and pressure P.

In the process of establishing an expression for $S_{bh}$, Bekenstein performs a thought experiment regarding a single particle falling into a black hole. Then he states “*Of course, only such an elementary particle can be regarded as having no internal structure. Therefore, the loss of information associated with the loss of such a particle should be minimum. And indeed we find that the increase in black-hole entropy is smallest for just such a particle. This supports our view that* 2ℏ *is the increase in rationalized area associated with the loss of one bit of information. Following our program we shall equate the minimum increase in black-hole entropy,* ${\left(∆S_{bh}\right)}_{min}=2\hbar df/d\alpha$*, with* ln2 *the entropy increase associated with the loss of one bit of information*”.

It is not necessary to mention here the final expression for entropy devised by Bekenstein. It is worth mentioning though, that he uses a version of the classical thermodynamic expression:

(21) $$T^{-1}={\left(\partial S/\partial E\right)}_{V},$$

adapted to black hole parameters to arrive at a black hole temperature $T_{bh}$. Then, he states (his emphasis) “*But we emphasize that one should not regard* $T_{bh}$ *as the temperature of the black hole; such an identification can easily lead to all sorts of paradoxes, and is thus not useful*”. Also, in the text he states “*it should be clear that the black-hole entropy we are speaking of is not the thermal entropy inside the black hole*”. As a matter of fact, after the 1974 Hawking’s manuscript, physicists considered them as equivalent and this resulted in more than 50 years of black hole information paradox.

### 4.2. Quantum States as Information

Some physicists may argue that Hawking was right in his 1974 manuscript, that indeed one can attribute thermodynamic entropy and temperature to a black hole based on information physics. We will demonstrate that the issue goes even deeper, and that there is no basis for such a claim.

It is clear from the 1973 Bekenstein’s manuscript and from the next ones that follows in the field, that he considers that each quantum state can be associated with one bit of information, being this information applicable to Shannon’s model. This stance is held by a large group of physicists nowadays, and it was epitomized in a rather radical form by Bekenstein’s advisor J. A. Wheller, by the phrase “it from bit” (for a detailed discussion on this matter, see [22]). We claim that this stance is unsubstantiated if we follow the principles of Shannon, Landauer and even Brillouin.

Firstly, we argue that Brillouin demonstrated that not every quantum state can be considered as information; he expresses that in his gas expansion example as we discussed in Section 3.4. Moreover, when he examines the measurement problem, he adds the α factor indicating that every measurement requires much more than the minimum energy, denoting that one bit of information is constituted by a set of many quantum states. This is, in fact, the usual case, that information is conveyed in such a way that each bit comprises a very large set of quantum states. We know for a fact that real computing devices operate several orders of magnitude above the Landauer limit [10].

However, let us analyze this issue from another perspective. In Bekenstein’s thought experiment of a particle falling into a black hole, he considers the particle as a message with one bit of information, related to the question “*does the particle exist or not?*” and, according to him, the answer is known to be yes. Known by whom? This is a very fundamental question. In this thought experiment, the only being that knows about the existence of the particle is the being thinking about the experiment in this specific way. Brillouin defines the information in the mind of an individual as free information, the kind that is not subjected to a connection to negentropy, i.e., connection to thermodynamic entropy. In a practical situation, the only way of knowing about the existence of the particle would be to measure it. If the particle is detected before the event horizon it would not fall inside the black hole.

We should analyze Bekenstein’s thought experiment in terms of theory of communication. According to Shannon’s sketch in Figure 1, there is a source of the message, a transmitter, a receiver, and a destination. Is a black hole a valid destination? What is the protocol defining the variables of communication? Morse code? English grammar? We have to suppose there is a protocol that establishes that detecting a particle means “this particle exists”. It could mean a different thing in another protocol. Detecting a single particle could be just noise in a third protocol. If we let ourselves enable Bekenstein’s thought experiment, we can try to devise a situation in which a scientist sends a particle to the black hole, and there is a protocol establishing that, the detection of the particle means “yes”. Still, this situation does not suffice to fulfill Shannon’s and Landauer’s requirements. After all, it is a yes-or-no bit of information, therefore, we are left with the question, what is the “no” state in this communication? In a communication system there should be a variable with a state for “yes” and another state for “no”. In the computation process, in order to guarantee a proper signal-to-noise ratio, there should be a switching mechanism, that correlates the “yes” and “no” states via an energy barrier $U_{b}$ according to Landauer’s principles (Figure 2a, or a proper separation enabled by a long diffusion process according to Figure 2b). The energy dissipated in this switching corresponds to one half of the hysteresis loop associated with the memory unit associated with the measurement system. We believe there is no mechanism possible that reconciles a communication process conceived by Shannon, according to energetic rules established by Landauer, to Bekenstein’s thought experiment. We conclude that a single quantum state is hardly a bit of information. In the end, in the same way that happened with the paradox posed by the Maxwell Demon, the solution to the black hole information paradox relies on the measurement process by a receiver.

Messages in Sumerian language written thousands of years ago are still very well readable nowadays in clay tablets. Each sign in cuneiform is still very stable and measurable because it consists of a stable solid system of ~10^24^ particles. Modern information systems fall in the logic of energy efficiency, lower costs, and Moore’s law, but still, memory technology engineers struggle to maximize the internal area of the corresponding hysteresis loop in order to maximize data readability and stability. This is a trade-off engineering problem in the course of miniaturization. As we discussed in previous sections, we should use hysteresis loops to analyze memory devices in a similar way we use thermodynamic cycles to discuss engines. A single quantum state memory is hardly a memory device in the analogous manner that a single particle in a piston–cylinder system is hardly an engine. The corresponding cycles in each case would yield a vanishingly small internal area, if any. Moreover, one could define a communication protocol in which the whole black hole thermodynamics is not related to information, but just to noise.

Finally, that does not mean that we cannot calculate entropy by counting the internal configurations of a system in terms of its quantum states. We can resort on Boltzmann entropy that was established before Shannon entropy with no connection to information. However, in the case of black holes, this has not yet been established as we are going to see in Section 4.4.

### 4.3. Black Holes Inside a Piston–Cylinder System

We already demonstrated that information science has not been adequately used in black hole physics. Now we discuss whether it is plausible to use the same thermodynamic principles devised for the cylinder–piston system to describe the behavior of black holes.

After the establishment of the black hole thermodynamic with the Hawking radiation, Bekenstein [23] and Hawking [24] published manuscripts in which they restated their main conclusions on temperature and entropy of a black hole and discussed the relationship between thermodynamics and black holes even further.

Hawking, for instance, in his work published in 1976 entitled “*Black holes and Thermodynamics*” concludes that black holes present negative specific heat and that the canonical ensemble is not suitable to model systems with black holes. In this case, it is reasonable to consider such systems as very special cases in thermodynamics. Thus, it is questionable the validity of traditional thermodynamic expressions such as Equation (21).

In thermodynamic analysis, there are very fundamental aspects to be considered, such as defining the boundaries of the system, its volume, and how it interacts with the environment. Very often, the volume of the system is defined by a container, with specific qualities such as adiabatic, rigid, permeable, etc. However, it is remarkable that Bekenstein and Hawking would place black holes inside containers in a very paradigmatic way. For instance, in 1975 Bekenstein published a manuscript called “*Statistical black-hole thermodynamics*” [23]. In his analysis trying to establish a generalized maximum-entropy principle he states “*Let us illustrate this principle by considering the problem of a Kerr hole formed by collapse which is enclosed in a container with walls that perfectly reflect all the relevant radiations.*” In his 1976 manuscript, Hawkings does something similar while arguing that black holes should be modeled using microcanonical ensemble when he states “*Consider, for example, a certain amount of energy E placed in an insulated box of volume* V*. Assume, for simplicity, that this energy can be distributed only among gravitons and black holes*”. We have to take into account that the parameter “V” appears in the next expression for the system’s temperature. We have to recall our discussions regarding Figure 6 and Szilard’s engine, that very often, statistical mechanics offer poor definitions of a wall and how it interacts with the system. If the Szilard’s engine can be considered already far removed from a real physical system, we can argue that the systems envisaged by Hawking and Bekenstein of black holes inside insulating or perfectly reflective boxes are even farther removed. How can we justify the presence of insulating walls surrounding astrophysical systems?

### 4.4. Black Hole Entropy in the Current Literature

Some physicists may question whether the criticism raised by this manuscript still holds given the current situation in black hole thermodynamics. Is the Bekenstein–Hawking entropy equation still prevalent in the field? Has the equation been derived from different, more solid principles?

In simple notation, the Bekenstein–Hawking entropy formula may be expressed as:

(22) $$S_{BH}=\frac{A}{4G}\;,$$

where A is the area of the event horizon, and G is Newton’s constant [25].

A very recent review on black hole thermodynamics [26] celebrated the 50th anniversary of this equation. One of its main focuses is “*the information paradox that encapsulates a deep conundrum between gravitation and quantum physics*”, reinforcing the widely accepted fact that a consistent quantum theory of gravity is missing. Another recent review, this time specifically on Bekenstein–Hawking entropy, classifies Equation (22) as “*a cornerstone of modern theoretical physics, with implications that continue to reverberate through contemporary research*” [27]. Another recent publication in the area states “*This remarkable formula is universal*” [25]. It is worth noticing that this publication was praised by a Science news article entitled “*Have scientists finally made sense of Hawking’s famous formula for disorder in a black hole?*” [28]. The news emphasizes that the work published by Balasubramanian aimed to calculate the black hole entropy using Boltzmann’s definition of entropy. As the article states, “*So far, they’ve been able to do that only for unrealistic black holes using speculative string theory—which assumes every fundamental particle is a tiny string or multidimensional “brane”*”, and later on states that the publication has been received with strong criticism. This view on the status of Equation (22) has been rather constant over time [29,30].

Despite its wide acceptance, the general validity of Equation (22) has been questioned before [31]. Over the decades, many physicists tried to gain better understanding of black hole entropy under deeper principles. One of the most praised models in this endeavor is known as Holographic Principle [32]. However, none of the tentative approaches achieved wide acceptance due to several reasons, such as the reputation decline of string theory or questionable assumptions, many of them raised in Section 4.2 and Section 4.3 such as confining astrophysical systems in very specific and unrealistic boxes, and usage of inadequate statistical ensembles [29,33].

### 4.5. The Connection Between Entropy and Gravity

Most physicists believe that we do not have a rigorous definition of entropy for systems coupled to gravity [34]. In the latest decades, the statistical mechanics for cosmological systems have been dominated by thermodynamics of horizons. We hope to have demonstrated that this approach, apart from being limited to back holes, has fundamental shortcomings.

The main issue regarding a gravitational gas cloud system is that it is not confined inside a box of volume V just as a traditional thermodynamic system, and we may ask how we can circumvent this matter.

Let us consider an intermediary case. Our atmosphere is not confined in a box in a classical way. It is bound by the ground at the bottom and by the gravitational force of the Earth at the top. It is the interaction between the particles that maintains the pressure that holds an inhomogeneous layer of gas that extends to ~10^4^ m in altitude. Particles of a gas do interact with each other. In fact, the mean free path of our atmosphere is around 10^−8^ m. On the other hand, the ideal gas equation gives useful results for pressures up to 10 atmospheres. However, while we arrived at Equation (11) following the steps put forward by Reif, we ignored the interactions between the particles. There is a fundamental issue here that we have to take into account.

In the process of calculating the partition function (Ω) of a thermodynamic system (as we did in a previous section), we evaluate the configurations in the phase space of the conjugate variables position (q) and momentum (p) of the particles of the system. Often, as we did by following Reif’s steps, we associate p with the kinetic energy K to evaluate χ(p). However, as the interaction between the particles U have been disregarded, the possible states for q are directly associated with the volume V of the container in order to evaluate φ(q). As already stated, the container is poorly modeled and it is implicit in the assumptions that the particles “know” that they are confined in a volume V. Our argument is that the particles of the gas can only be subjected to the effects of interaction potentials. In a rather large box of a gas with pressure and temperature similar to our atmosphere, the most probable interaction is particle–particle when compared to particle–walls interactions. If the volume of the box is changed, the interactions between particles will be changed and adjusted very rapidly and accordingly.

If we consider a gravitational gas cloud, preferably a rather small one consisting of N particles, where relativistic effects can be disregarded, the problem could be classically treated without the necessity of modeling the walls of a container. This has already been done [35]. As usual, the variable p has been associated with the kinetic energy *K* to evaluate χ(p). Furthermore, the variable *q* has been associated with the interaction between the particles U order to evaluate φ(q) resulting in an effective volume V for the partition function calculation. This resulted in the following expression for the entropy of a gravitational gas cloud system:

(23) $$S\left(K,\;U\right)=k\ln B+\frac{3N}{2}k\ln\left(2mk\right)-3Nk\ln\left(-\frac{U}{C_{0}}\right),$$

where B and $C_{0}$ are constants. Our purpose here is just to show that there are proposals for formal connections between gravity and entropy that are not related to information physics. For the process of deduction of Equation (23), see reference [35].

## 5. Conclusions

As they focused on communication systems and computation processes, the works of Shannon and Landauer are consistent. Shannon sustained that information entropy was only an analogy to thermodynamic entropy.

We demonstrated that the works by Szilard failed to establish a direct connection between information and thermodynamics.

When Brillouin tried to generalize the principles of information entropy to general physics, some foundational principles in the theory of communication were neglected. The two examples brought forth by him to demonstrate the direct relation between information and entropy befell into the problem of measurement. This resulted in a paradoxical situation with the information bearer, that is akin to the problem found in Szilard’s work. Moreover, even Brillouin maintained that not every quantum state (or internal configuration) of the system can be directly linked to one bit of information.

The black hole entropy and temperature were calculated by Bekenstein and Hawking relying on Shannon’s formula for information entropy. As we demonstrated, this procedure is unfounded for several reasons. First, they rely on the invalid notion that there is a direct correlation between one bit of information and a single internal configuration. Specifically, the thought experiment put forward by Bekenstein, of a particle falling into a black hole, is invalid, thus failing to establish the direct connection between one internal configuration of a system to one bit of information in the black hole realm. As Brillouin did in his gas expansion example, Bekenstein also failed to include a measurement process, resulting in the paradox of the information bearer. Furthermore, in any communication system, there should be a protocol that establishes a set of variables and a measurement process that guarantees data readability and stability. In the computation process, in order to guarantee a proper signal-to-noise ratio, there should be a switching mechanism, to distinguish between variables. The energy dissipated in this switching process corresponds to one half of the hysteresis loop of the memory unit associated with the measurement system. This is the essential connection between energy and information. Thus, the models elaborated by Shannon and Landauer are not reconcilable with black hole thermodynamics.

Finally, a solution to the black hole information paradox is presented, once we take into account that internal configurations of a system is not the same as information. If an apple falls into a black hole, we have to analyze it as it is, for an apple is hardly a message.

## Data Availability

No new data were created
